# Toward an Emotional Individual Motor Signature

**DOI:** 10.3389/fpsyg.2021.647704

**Published:** 2021-05-31

**Authors:** Juliette Lozano-Goupil, Benoît G. Bardy, Ludovic Marin

**Affiliations:** EuroMov Digital Health in Motion, Univ Montpellier, IMT Mines Ales, Montpellier, France

**Keywords:** emotion, movement, individual motor signature, mirror game, kinematics

## Abstract

Bodily expression of felt emotion has been documented in the literature. However, it is often associated with high motor variability between individuals. This study aimed to identify individual motor signature (IMS) of emotions. IMS is a new method of motion analysis and visualization able to capture the subtle differences in the way each of us moves, seen as a kinematic fingerprint. We hypothesized that the individual motor signature would be different depending on the induced emotional state and that an emotional motor signature of joy and sadness common to all participants would emerge. For that purpose, we elicited these emotions (joy, sadness, and a neutral control emotion) in 26 individuals using an autobiographical memory paradigm, before they performed a motor improvization task (e.g., the mirror game). We extracted the individual motor signature under each emotional condition. Participants completed a self-report emotion before and after each trial. Comparing the similarity indexes of intra- and inter-emotional condition signatures, we confirmed our hypothesis and showed the existence of a specific motor signature for joy and sadness, allowing us to introduce the notion of emotional individual motor signature (EIMS). Our study indicates that EIMS can reinforce emotion discrimination and constitutes the first step in modeling emotional behavior during individual task performances or social interactions.

## Introduction

Emotions produce transient changes at cognitive, physiologic, and behavioral levels for individuals who experience it (Scherer, 2001). Behavioral responses include changes in facial expressions, voice tonality, posture, and gestures. Ekman (1992) described six biologically based emotional facial expressions, innates, universals, and easily identifiable (i.e., anger, disgust, surprise, happiness, fear, and sadness). However, emotions are recognized not only from facial expressions but also from whole body posture, gestures, and movements, even in the absence of a facial expression (de Meijer, 1989). Numerous studies demonstrated that participants successfully identified emotions from watching other people’s movement, especially from professional actors and dancers (de Meijer, 1989; Montepare et al., 1999; Sawada et al., 2003). For instance, Sawada et al. (2003) recorded dance with arm movement under joy, sadness and anger, and asked volunteers to identify the expressed emotion, reporting significant identification rates for each emotion. Affective states can also be extracted from recordings reach and grasp actions (Pollick et al., 2001). These studies allow us to build an understanding of specific motor features corresponding to emotion types. For example, movements performed with sadness have been characterized as having a collapsed upper body, low dynamics (Wallbott, 1998), and very smooth, loose, slow, and soft moves (Montepare et al., 1999). Camurri et al. (2003) added low rhythm and a weak tension in movement to the characteristics of sadness. Conversely, joyful movements were found to be characterized by elevated shoulders, backward head posture, high dynamics (Wallbott, 1998), and as gestures moving away from the center of the body (Camurri et al., 2003). In addition to these general movement markers, more specific postural features have been proposed to discriminate emotional categories. For instance, joint angles, vertical movements and symmetry to the longitudinal axis help to distinguish joy from the neutral state (Lopez et al., 2017). Finally, more distal information coming from trunk, arm, and head position are also major cues for characterizing expressed emotion (de Meijer, 1989).

While these studies strongly suggest that emotions are associated with detectable differences in body movement patterns, they do not allow to control for whether the emotion were felt and not only portrayed. In fact, as emotions can be acted by professional actors, they can be also successfully induced in a laboratory setting with pictorial stimuli (Lang, 2005), films (Gross and Levenson, 1995), music (Västfjäll, 2001), or autobiographical recall (Strack et al., 1985). Siedlecka and Denson (2019) recently reviewed the experimental methods for inducing basic emotions, classifying their efficacy according to evoking emotions. Visual methods and autobiographical recall effectively induce the six basic emotions, while music induces anger, fear, and disgust with greater difficulty (Ekman, 1992; Siedlecka and Denson, 2019). Crane and Gross (2007) induced sadness, joy, and anger to participants with autobiographical recall while asking them to walk a distance of 5 m. Kinematic analyses showed that gait was different according to the emotions induced, particularly walking speed. The idea that body and emotion are interconnected comes from the theory of embodiment, which has received support from several branches of psychology and cognitive sciences (Gallese, 2005). Embodiment denotes the theoretical perspective that representations and cognitive operations are fundamentally anchored in their physical context (Niedenthal et al., 2005). According to Barsalou (2009), every cognitive activity, every emotion and affect have a sensorimotor component. The embodiment theory allows to explain these abovementioned studies by the emotion-specific repercussion of the psychological response onto bodily expression. This two-way dialog has also justified the apparition of specific emotional states and feelings visible in adopting particular postures and movements (Riskind, 1984; Stepper and Strack, 1993). The field of embodied cognition has been well documented, but more motor and behavioral studies are still needed to support this approach.

For this purpose, authors have developed several movement analysis methods, first qualitative and then quantitative, yet a universal method specifying how and what type of emotion are embodied is still missing. For instance, Castellano et al. (2007) measured the quantity of motion, computed with a technique based on silhouette motion images. Changes in momentum and in the hand-head-hand triangle have also been calculated from videos (Glowinski et al., 2008). The Laban Movement Analysis is another well-established system for describing body movement (Amighi et al., 1999). More recently, Dael et al. (2012) created a systematic movement analysis method: The Body Action Coding System, which focuses on muscle activation patterns during the perception and expression of different emotions (Dael et al., 2012). These studies used different movement analysis methods to describe the associations between movement and emotion, preventing a thorough comparison of their results. While the majority of these studies tend to demonstrate kinematic or postural changes as hallmarks of emotional arousal, another interesting method is the extraction of individual motor signature (IMS). This technique is based on the existence of a person-specific motion signature, a sort of kinematic fingerprint. It uses velocity as key kinematic feature to analyze movements (Słowiński et al., 2016). In theory, the IMS is supposed to be largely stable across time (invariance) and different from those of others (distinctiveness). Studies have shown that every individual owns their proper individual motor signature, i.e., their idiosyncratic way to move in a neutral state (Słowiński et al., 2016; Coste et al., 2020). IMS can be represented by ellipses in a similarity space, an abstract two-dimensional geometrical space calculated using dimensional reduction techniques and minimizing distances between trials and individuals (see section “Materials and Methods” for details). These ellipses can be large or small depending on the spread of the data points for each individual and provide information on intraindividual variability. The distance between the ellipses reports disparities in IMS, which allows discrimination between individuals. Importantly, it is the interaction between the individual and the environment that the task brings out as a motor signature (Coste et al., 2019). Based on the previous evidence that emotions produce cognitive, physiologic, and behavioral responses (Scherer, 2001), we might assume that emotions such as joy or sadness might channel some universal characteristics onto the motor signature of an individual, much alike task constraints, which can modulate IMS in comparison with the emotionally neutral behavioral state. As the change in motor behavior was previously found to be identifiable by the naked eye and computer algorithms (Camurri et al., 2003), we therefore focused our analysis on the investigation of whether it can be distinguished with IMS.

In the present study, we hypothesize that there is specific individual motor signature for joy and sadness common to all participants. We assume that both IMS extracted from joyful and sad movements will be dissimilar from the neutral IMS and between them, as their movements’ velocities are different. This study therefore contributes to the movement-emotion literature in two important ways. First, it will take further the understanding of emotion expression and recognition. Second, it provides a starting point to connect research in psychology and in biometrics, allowing the possible detection of different emotional states of moving individuals.

## Materials and Methods

### Participants

Twenty-six participants, including 18 females (mean age = 25.6 years, *SD* = 7.1 years) and eight males (mean age = 21.3 years, *SD* = 0.4 years) participated in the study. None reported any history of psychiatric or neurological disorders, and they had no history or current use of psychoactive medication. None of the participants reported the presence of disorders that could affect the creation of movement with their dominant arm. The sample has been recruited from every educational level through announcements on the EuroMov DHM laboratory website, the department of Human Movement Sciences of Montpellier website and in the community. All subjects gave their written informed consent. All procedures were in accordance with the 1964 Helsinki declaration and were approved by the EuroMov Institutional Review Board (IRB # 2004A).

### Emotion Elicitation

One negative emotion (sadness) and one positive emotion (joy), in addition to the neutral emotion, were elicited by means of the autobiographical memory paradigm (Healy and Williams, 1999). Prior to data collection, each participant completed a worksheet with the task of describing a time from their own life when they felt a specific emotion. The instructions were to read the worksheet and to complete the following information: “Please describe in detail the one situation that has made you the happiest/saddest you have been in your life, and describe it such that a person reading the description would feel the happiness/sadness just from hearing about the situation.” These instructions were identical to those used in previous studies (Labouvie-Vief et al., 2003; Roether et al., 2009; Mills and D’Mello, 2014). The neutral emotion was elicited by writing about an everyday situation, which was not associated with any particular emotional arousal (Gross et al., 2012). In order to perform this task, participants were alone in a quiet room, seated at a desk, and typed their responses in a text box. They were told that the information provided on the worksheet was for experimental use only. There was no specific time limit for writing. To validate that the emotion was actually felt by the participant and not just portrayed, participants rated the intensity of their emotion felt using a questionnaire after the elicitation task. This questionnaire included the two target emotions, joy (“I felt glad, happy, and joyful”) and sadness (“I felt sad, down-hearted, and unhappy”), as well as two nontarget “distractor” emotions, surprise (“I felt surprised, amazed, and astonished”) and disgust (“I felt disgusted, repulsed, and revolted”). The two nontarget emotions were selected based on their similarity, in terms of valence and intensity, to the target emotions and were used as control conditions, decreasing the odds of selecting the measured emotion by chance. A five-item Likert scale (0, not at all; 1, a little bit; 2, moderately; 3, a great deal; and 4, extremely) was used to score intensity. All trials within a particular emotion condition for each participant were aggregated to create a single average emotion score.

### Experimental Task

**FIGURE 1:**
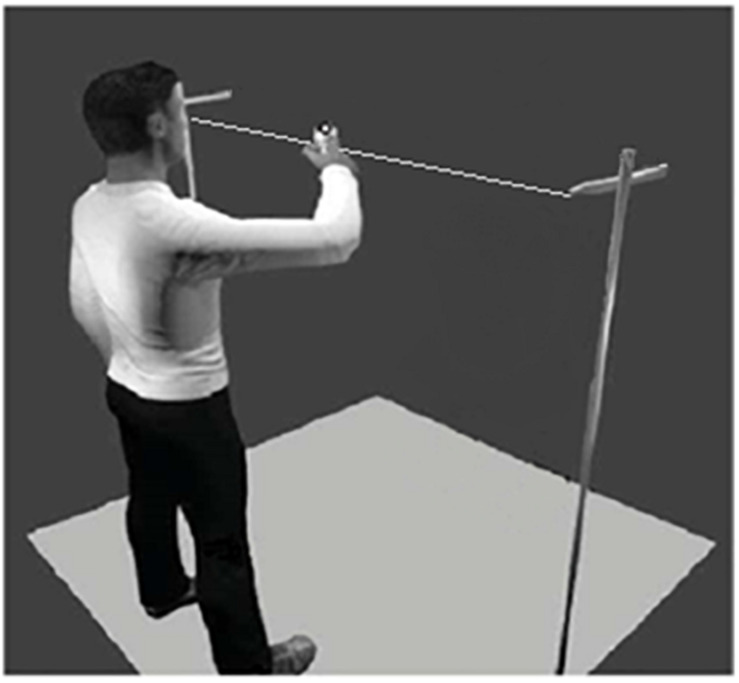
Experimental device showing one participant engaged in the improvizational motor task (i.e., the adapted Mirror Game, cf. Noy et al., 2011).

Participants were asked to create movement in an improvizational task, adapted from the mirror game paradigm (Noy et al., 2011), with their dominant arm in the horizontal axis. This task allowed participants to create rhythmic movements with different amplitudes, speeds, and frequencies. In this study, we used the same apparatus as the one used by Gueugnon et al. (2016) in which a thin plastic thread of 180 cm is suspended horizontally at 140 cm from the ground. There was a marker on a handle that could be freely moved along the thread, with very limited friction force (Figure 1). Movements of the marker were recorded with an optical motion capture (Vicon Motion Systems, Oxford, England), equipped with eight cameras. The three-dimensional trajectory of the marker was tracked with a spatial accuracy of 1 mm at a sampling rate of 100 Hz. After data acquisition, data were postprocessed using the Nexus 1.8.5 software to remove any missing data point below 5 frames.

### Experimental Procedure

Upon arriving at the laboratory, participants signed an informed consent form approved by the EuroMov Institutional Review Board and completed the Positive and Negative Affect Schedule (PANAS; Watson et al., 1988) in order to assess their emotional state prior to the experiment. Then they were asked to fill in the autobiographical recall worksheets for each of the specific emotion, then to fill in the questionnaire to measure their actual emotion and then to stand in front of the mirror game apparatus. When standing in front of the thread, they moved the marker along the thread with their dominant hand. The instructions were to “create complex, varied, and interesting movements” (Noy et al., 2011). They were encouraged to “be in the memory” and to “let the feelings from that time flow through the body” during every trials of the block, as in Gross et al.’s 2012 study. Prior to the experiment, participants were familiarized with the task and subsequently performed a test trial (30 s). These improvizational movements allowed the participants to express themselves and the experimenter to observe the emotional influence on their movements. Then they choose the distance between them and the thread; however, once placed, they were instructed not to move their feet or change their hand until the end of the condition. For each new trial, they were asked to put the marker in the center of the thread. After each condition, the questionnaire was filled out again to verify that the target emotion was still felt correctly. After the questionnaire, participants were instructed to try to forget their memory, and they were given at least 1 min of rest to do so before filling the next worksheet, as in the study of Fawver et al. (2014). The improvization task (i.e., Mirror Game) consisted of blocks of nine trials lasting 30 s each for the three conditions (i.e., neutral, joy, and sadness). This makes a total of 27 trials (9 trials ^∗^ 3 conditions). Each trial was separated by a 10-s break to allow participants to release the handle and get rest. The order of emotions induced was counterbalanced for all participants, only the neutral emotion was induced first. After the completion of all emotion blocks, participants were fully debriefed.

### Data Analysis and Statistics

For emotion induction, subjective recall ratings were analyzed using a Kruskal-Wallis one-way ANOVA for each question (“I felt glad, happy, and joyful,” “I felt sad, down-hearted, and unhappy,” “I felt surprised, amazed, and astonished,” and “I felt disgusted, repulsed, and revolted”) and for both measures (before and after the motor improvization task). For significant main effects, pairwise Wilcoxon tests were conducted with Bonferroni corrections. This analysis allows to ensure that the target emotion (joy and sadness) was felt with greater intensity when induced than the others (Fawver et al., 2014), i.e., that sadness and joy were more felt in sad and joy conditions, respectively.

Concerning individual motor signatures, we extracted them from the marker position in the *y* dimension for each participant and for each condition. The method proposed by Słowiński et al. (2016) was rigorously followed. Collected data were processed with Matlab R2014b (The MathWorks, Natick, MA). A zero-phase forward and reverse digital second-order lowpass (10 Hz cut-off) Butterworth filter was used. We cut out the first and last 1 s of the signal in order to avoid the inclusion of any transient behavior due to possible movement anticipation. The position time series were then used to numerically estimate their corresponding velocity time series. To differentiate position timeseries, we used a fourth-order finite difference scheme. Furthermore, we limited velocities to 10 m s^–1^ in the experiment (higher velocities were considered results of noise in the collected data). To analyze the movements of each participant and start the extraction of IMS, we calculated for each trial the probability density function (PDF) of the participant’s velocity, i.e., *velocity profile*. Each PDF is a normalized histogram of the velocity time series with 101 equally distant bins between −10 and 10 m s^–1^. Further details about data processing can be found in Słowiński et al. (2016).

**FIGURE 2:**
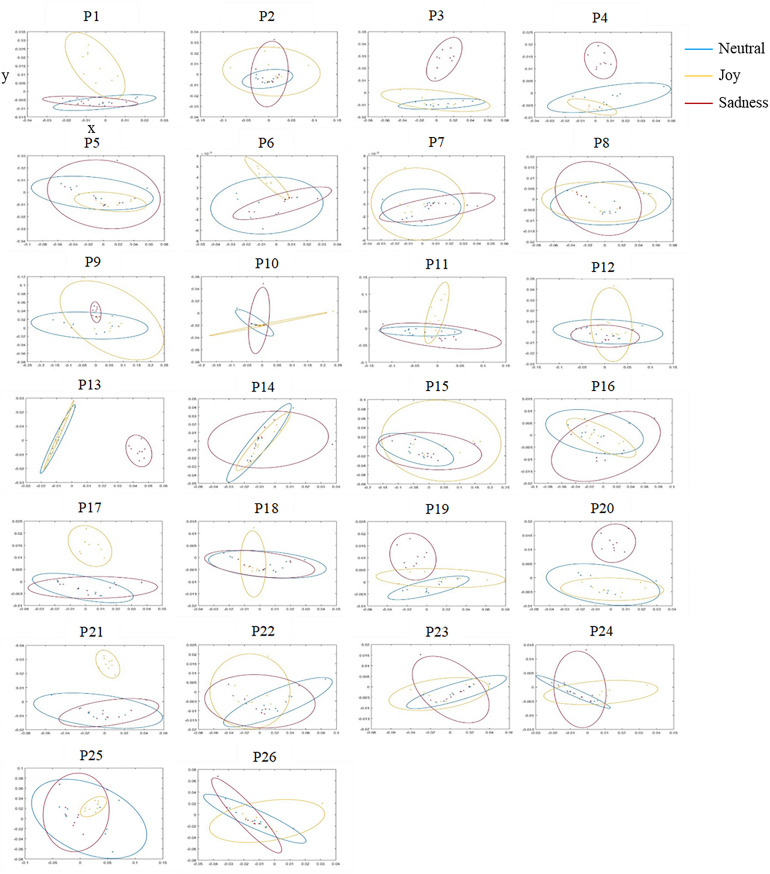
Individual motor signatures (IMS) of each participant in the similarity space, for the three tested emotional conditions neutral (in blue), joy (in yellow), and sad (in red). The nine small dots in each ellipse correspond to the nine individual trials by emotional condition. *X*-axis and *Y*-axis represent the first two dimensions of the multidimensional scaling analysis (MDS), proper to each participant (see text for details).

In order to compare the PDFs of the velocity signals between them, we used the Earth Mover’s Distance (EMD). The EMD is a recognized mathematical tool for studying nonlinear phenomena (Muskulus and Verduyn-Lunel, 2011) and is a measure of similarity between two histograms. Conceptually, this metric is based on the principle of minimum work that must be provided to transform one histogram into another. It therefore depends on the distance and the quantity to be transported. The greater the amount of work, the less the compared distributions are similar, this value then moves away from 0. Conversely, the more EMD tends toward 0, the more the distributions are similar. Empirically, the EMD is the area of the difference between the cumulative density functions (CDFs) of each distribution, i.e., the cumulative sum of the intervals of the standardized histogram. We gathered every distance between each PDF to create a distance matrix, and we used the multidimensional scaling analysis (MDS; Pollick et al., 2001; Giese et al., 2008; Słowiński et al., 2016) to represent the similarity of the participants’ signatures visually as 2D maps. The MDS analysis allows to reduce the dimensionality of the data and visualize relations between the objects under investigation while preserving as much information as possible. In this abstract space, called the *similarity space*, small dots correspond to individual recordings and each ellipse indicates the 0.7 mass of the bivariate normal distribution fitted to those dots. Thus, the ellipses represent the IMS for joy, sadness, and neutral (see Figure 2 for IMS of all participants).

**FIGURE 3:**
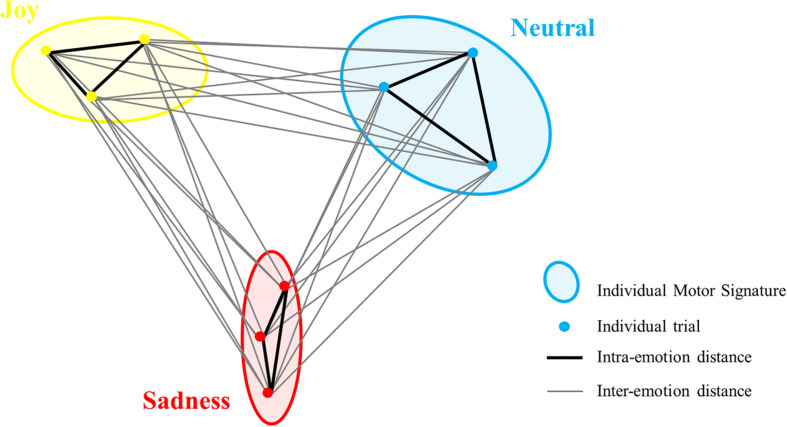
Schematic example of ellipses of the three conditions (IMS) with intra-emotion distances (in black) and inter-emotion distances (in gray) for one participant. To compute the intra-emotion similarity index, the mean distance in black was calculated for each condition. To compute inter-emotion similarity index, the mean distance in gray was calculated between each condition. For clarity, the interaction of only three trials per condition is displayed (represented with dots), instead of nine trials in the actual experiment.

Finally, we used the values from the distance matrix to compute the motor signatures in the three conditions (neutral, joy, and sad). We computed the average distances between each participant’s trials for each emotional condition to obtain three inter-emotional similarity indexes for each participant: neutral/joy, neutral/sad, and joy/sad. Intra-Emotional similarity indexes for neutral, joy, and sadness were also computed for each participant from the distance matrix (neutral/neutral, joy/joy and sadness/sadness; see Figure 3 for a schematic example). Similarity indexes varied from 0 to 1 representing the most similar to the least similar IMS, as they revealed distances between velocity profile of each trial/signature. Intra- and inter-emotion similarity indexes were compared for the three conditions, in order to find low similarity in trials from different emotions and high similarity in trials of the same emotion.

Due to violations of the normal distribution, we conducted three one-sample Wilcoxon signed-ranks tests to verify whether inter-emotional similarity indexes were higher than 0, and we calculated three Friedman tests for intra- and inter-emotional similarity indexes. A final Friedman test on intra-emotional similarity indexes only was conducted to examine whether interindividual variability was reduced for the emotional conditions (joy and sadness) relative to the neutral condition. Statistics were calculated using R software and alpha was set to 0.05 for all statistical tests.

## Results

### Emotion Elicitation

Before and after each block of nine trials, participants completed a questionnaire assessing their intensity of felt emotion on a scale from 0 (not at all) to 4 (extremely). All response averages for the intensity questionnaire are presented in Table 1. Concerning the first measure (i.e., before each motor task), Kruskal-Wallis ANOVAs indicated a significant main effect for condition on each question (all *p* < 0.001) except for “I felt surprised, amazed, and astonished.” For the main effect of condition on “I felt glad, happy, and joyful,” *H*(2) = 33.70, *p* < 0.001, post hoc tests revealed that participants reported a significantly higher intensity during the joy condition compared with sadness and neutral conditions. Participants responding to “I felt sad, downhearted, and unhappy,” *H*(2) = 37.16, *p* < 0.001 reported a significantly higher intensity during sadness condition compared with joy and neutral conditions. Finally, for the main effect on “I felt disgusted, repulsed, and revolted,” *H*(2) = 32.79, *p* < 0.001, post hoc analyses revealed that participants reported a significantly higher intensity during sadness condition compared to joy and neutral conditions. Overall, our results indicate that participants felt the target emotions (joy and sadness) in the corresponding condition with more intensity than in the other conditions during the recall procedure.

**TABLE 1:** Mean scores for each question by emotional condition before and after the mirror game (MG).

	**Joy**		**Sadness**		**Neutral**	
**Measurement time**	**Before MG**	**After MG**	**Before MG**	**After MG**	**Before MG**	**After MG**
I felt glad, happy, and joyful	3.65	3.04	1.00	1.54	2.35	1.96
I felt sad, downhearted, and unhappy	0.35	0.11	2.62	2.0	0.46	0.50
I felt surprised, amazed, and astonished	0.46	0.54	0.31	0.23	0.81	1.42
I felt disgusted, repulsed, and revolted	0.08	0.08	1.69	0.81	0.15	0.12

*Mean scores for each question by emotional condition.*

Concerning the second measure (i.e., after each motor task), Kruskal-Wallis ANOVAs indicated a significant main effect for condition on the two questions of interest (*p* < 0.001). For the main effect of condition on “I felt glad, happy, and joyful,” *H*(2) = 17.7, *p* < 0.001, post hoc tests revealed that participants reported a significantly higher intensity during joy compared with sadness and neutral. Participants responding to “I felt sad, downhearted, and unhappy,” *H*(2) = 32.1, *p* < 0.001 reported significantly greater intensities during sadness compared with joy and neutral. Altogether, these results indicate that participants felt the target emotions (joy or sadness) from the beginning to the end of the motor task.

Prior to the experiment, mean score of PANAS positive was 31.3 (*SD* = 4.60) and mean score of PANAS negative was 13.1 (*SD* = 3.20). The inclusion of PANAS scores as covariates at the individual level in the emotion elicitation analysis did not changed the results.

### Motor Signatures of Emotions

To investigate the evidence for impact of emotion on motor signatures, three one-sample Wilcoxon signed-ranks test were run. The outcomes indicated that posttest scores were significantly higher than 0, *Z* = 351, *p* < 0.001 for the three inter-emotional similarity indexes (i.e., neutral/sad, neutral/joy, and sad/joy), revealing that ellipses were significantly distinct from each other.

Then to compare intra- and inter-emotion similarity indexes, we conducted a Friedman test for each condition. For example, for the joy Friedman test, the levels were joy/joy, joy/sad, and neutral/joy. Means and medians of every intra- and inter-emotional similarity index are presented in Table 2. The Friedman’s test revealed a main effect of intra- and inter-emotional similarity for all conditions, *X*^2^_*F*_(2) = 21.3, *p* < 0.001, n^2^_*p*_ = 0.41 for Joy, *X*^2^_*F*_(2) = 21.5, *p* < 0.001, n^2^_*p*_ = 0.41 for sadness and *X*^2^_*F*_(2) = 8.38, *p* < 0.05, n^2^_*p*_ = 0,16 for neutral. Post hoc test using a Durbin-Conover test revealed that joy intrasimilarity indexes (joy/joy) were significantly lower than neutral/joy and joy/sad similarity indexes (both *p* < 0.001). Sadness intrasimilarity indexes (sad/sad) were significantly lower than neutral/sad and joy/sad similarity indexes (both *p* < 0.05). Finally, neutral intrasimilarity indexes (neutral/neutral) were significantly lower than neutral/sad similarity indexes (*p* < 0.001). As a low index means a high similarity, these results showed low similarity in trials from different emotions and high similarity in trials of the same emotion.

**TABLE 2:** Means and medians of intra- and inter-emotional similarity indexes for joy, sad, and neutral conditions.

**Similarity index**	**Joy/Joy**	**Sadness/Sadness**	**Neutral/Neutral**	**Neutral/Joy**	**Neutral/Sadness**	**Joy/Sadness**
Means	0.0210	0.0238	0.0291	0.0302	0.0372	0.0349
Medians	0.0155	0.0173	0.0236	0.0220	0.0262	0.0239

*An index close to 0 means high similarity. Conversely, an index that moves away from 0 means low similarity.*

Finally, a Friedman test on intra-emotional similarity indexes was conducted to examine whether interindividual variability was reduced for the emotional conditions (joy and sadness) relative to the neutral condition. The outcomes revealed a main effect of emotion *X*^2^_*F*_(2) = 11.4, *p* < 0.01, n^2^_*p*_ = 0.22. Post hoc test using a Durbin-Conover test revealed that neutral intrasimilarity indexes (neutral/neutral) were higher than joy intrasimilarity indexes (joy/joy; *p* < 0.001) and sadness intrasimilarity indexes (sadness/sadness; *p* < 0.05). These results showed a lower interindividual variability in joy and sadness condition relative to the neutral condition, highlighting the similarity of participants’ movements when they felt an emotion compared with when they did not.

Presentation of every emotional IMS in the ellipse form allowed to visualize intra- and inter-emotional similarity (see Figure 2). For example, when two ellipses are overlapping, similarity between them is high, as the movements corresponding (e.g., Figure 2, P8). Conversely, when two ellipses are distinct from each other, similarity is low so we can assume that movements produced during the mirror game were different according to conditions (e.g., Figure 2, P19). However, there were substantial interindividual variations between IMS of the three conditions. In particular, we noticed that some participants had more distinct ellipses for joy and other for sadness. Some mostly modified their movements during the joy condition while others during the sad one.

## Discussion

The aim of the present study was to highlight the motor signature of emotions during a very simple motor improvisation task. We successfully induced in 26 participants joy, sadness, and a neutral emotion and asked them to create movements in one dimension. We hypothesized that the participants’ motor signatures would be different depending on their emotional state and that an emotional motor signature of joy and sadness common to all participants would emerge.

Analyses of felt emotions showed that the task of inducing emotions through autobiographical recall was effective. The questionnaire of the felt emotions was carried out a first time just after the emotion induction task and a second time after the creation of the movements, in order to verify that the target emotion persisted throughout the motor task. The participants felt joy, sadness, and neutral during the motor tasks. Regarding the second measures, target emotions was significantly felt but intensity scores were lower than those obtained for the first measures. While some studies claim that the autobiographical recall technique can induce emotions over a period of 3 to 8 min (Braun et al., 2018), this duration is in fact highly variable. It depends on the event that triggers the emotion (duration and evaluation of the emotion), the emotion itself (nature and intensity of the emotion), and the person experiencing it (dispositions and measures to regulate emotion) (Verduyn et al., 2015). Lasting about 5 min, it is conceivable that our motor task allowed the emotion to dissipate a little bit. Moreover, no study, to our knowledge, using autobiographical recall has carried out a second questionnaire of emotions felt after the measured task as we did, and therefore the comparison with other studies is difficult.

As in the study by Mills and D’Mello (2014), incident emotions (i.e., emotions felt other than those expected experimentally) had also been induced during the autobiographical recall. Indeed, intensity scores of surprise and disgust emotions were higher than 0 (see Table 1). Some memories can then be simultaneously associated with several emotions of similar valence. These results question the principle of uniqueness between a stimulus and its emotional consequence from the discrete emotion model (Ekman, 1992). In short, our emotion induction method was found to be efficient, allowing us to draw conclusions from the data analyses. This supports the use of autobiographical recall in emotion experiments performed in the laboratory.

While many studies have focused on the discrimination of emotions through movements, modeling and recognition techniques are still blurred and the variability of results persists. Our IMS analysis allowed us to obtain similarity indexes within and between the motor signatures of the three different emotional conditions. These inter-emotional indexes being quite different from 0 and significantly higher than intra-emotional ones, we can affirm that the ellipses corresponding to neutral, joy, and sadness are well distinct in the similarity space and therefore we propose the term of emotional individual motor signature (EIMS) to capture them. In other words, ellipses represent different and independent motor signatures. Each ellipse is supposed to correspond to a particular style of movement (Słowiński et al., 2016; Coste et al., 2020), and we can therefore assert that participants adopted distinct motor behaviors when they felt a neutral emotion, a happy, or a sad one. Specifically, participants created movements with different velocities between the three conditions. Many studies have found that joyful movements are fast whereas sad movements are slow or slower (de Meijer, 1989; Atkinson et al., 2004; Crane and Gross, 2007; Roether et al., 2009). Our analysis supports the idea that there is a difference in arm velocity between these two emotions, but we add that the distinction between both emotional valences is not so obvious. This study relates to the *fingerprint hypothesis* of emotion, where the instances of each emotion category share a distinctive pattern of autonomic nervous system (ANS) activity and that different emotion categories have distinct fingerprints (Barrett et al., 2007; Gross and Barrett, 2011). Nevertheless, our results are also compatible with the *population hypothesis*, which claims that ANS patterns are highly variable within an emotion category and overlap with other categories (Barrett, 2013; Barrett and Russell, 2014). Indeed, from a motor point of view, ellipses representing the EIMS showed that there is still a substantial interpersonal variability within emotion and overlapping between emotions. This variability seems crucial to consider in motion-emotion literature, and the advantage of the IMS is to preserve everyone’s motor individuality while gathering by emotions.

Importantly, the emotional expressions captured in our study were not enacted as in the majority of studies in the area but felt by our participants. These results are in accordance with the embodiment theory, which considers that our cognitive and emotional activities must be understood in the context of our body expressions and our interaction with the environment (Niedenthal et al., 2005). They are totally intertwined. Gelder et al. (2004) used fMRI technology to show that the areas of the brain associated with emotion processing (e.g., the amygdala and the posterior cingulate cortex) and those dedicated to the representation of action and movement (e.g., the lower and middle frontal gyrus and the precentral gyrus in the primary motor cortex) are activated during a simple observation of pictures of body emotional expressions. It suggests that emotion is a combination of both neural and motor processes (Gelder et al., 2004). Thus, this study reinforces the embodiment theory on which our results are based and supports that an emotional state is manifested as a specific bodily expression.

Nevertheless, although participants felt sadness in the sad condition more than in the two others, the intensity score was low during the experiment. Despite that, the participants’ movements were significantly different in all three emotional conditions. These results support the idea of an unconscious part of the emotional process. Winkielman and Berridge (2004) showed participants’ subliminal emotional faces (neutral, happy, or angry) and gave them a novel drink to pour, consume, and rate. They also asked them to rate their current mood and arousal. As findings of this study report, conscious feelings were not influenced by subliminal presentation of emotional faces but participants’ consumption and rating of the drink were influenced by those stimuli. Thus, unconscious emotional reactions acted through basic biopsychological mechanisms that determine behavioral reactions rather than through cognitive mechanisms influencing interpretation of the stimulus (Winkielman and Berridge, 2004). In our study, participants who did not report feeling sad were still exhibiting changes in their movements compared with the neutral state, which means that a part of their emotional response was not conscious yet was reflected in their gestures. Furthermore, our findings might have had more pronounced main effect if we had compared joy and sadness with a neutral condition that is the same for everyone, as implemented in the other studies. It is essential to note that our study showed that neutrality, measured with IMS, is already an emotion in its current form, specific to each individual.

This study has methodologic limitations that must be considered. The movement used in this study was extremely simple and consisted in producing unidimensional improvizational movements of the hand in the lateral direction. Although this type of movement is restrictive, several other studies have used the mirror game to measure the link between synchrony and improvization (e.g., Gueugnon et al., 2016) to explore group dynamic (Himberg et al., 2018) and to define it as a sociomotor biomarker for schizophrenia (Słowiński et al., 2017). However, while our results strongly demonstrate the embodiment of emotional qualities in such as simple gesture, the generalization to more complex body movements remains an open question. The integration of a 2nd and a 3rd dimension in the IMS analysis would include more information about the movement, resulting in perhaps a better discrimination of each emotion. However, it requires an extensive mathematical analysis that still needs to be developed. This study is the first step to allow IMS analysis of more ecological emotional movements such as talking, walking, or dancing.

Finally, our results confirm that people change their behavior between several emotional states and adopt specific motor features (de Meijer, 1989; Montepare et al., 1999; Camurri et al., 2003). Those emotional behaviors can now be modeled, and we showed that there is a specific individual emotional motor signature for both joy and sadness, compared with each individual’s neutral IMS. We believe that our results open new perspectives on the analysis of human movement, extending the scope of the motor signature application, which can surely be adapted to other psychological processes such as motivation and self-esteem. This study improves the understanding of emotion expression and its quantification, for example, for patients with mental illnesses or behavioral disorders. Patients suffering with autistic spectrum disorder (ASD) can have impaired facial and bodily emotional expression (Loveland et al., 1994; Shalom et al., 2006), which contributes to the observed core social-communication disability that characterizes this disorder. Perhaps, extraction of EIMS from their motor behavior could be a novel pathway for indexing ASD in diagnostics and outcome measures of rehabilitation programs.

## Data Availability Statement

The datasets presented in this study can be found in online repositories. The names of the repository/repositories and accession number(s) can be found below: https://osf.io/byxaz/.

## Ethics Statement

The studies involving human participants were reviewed and approved by EuroMov Institutional Review Board (IRB # 2004A). The patients/participants provided their written informed consent to participate in this study.

## Author Contributions

JL-G developed the research idea with the help of LM and BB, and collected and analyzed the data under the supervision of LM and drafted the manuscript which was commented, discussed, and reviewed extensively by LM and BB. JL-G, LM, and BB developed the research design. All authors contributed to the article and approved the submitted version.

## Conflict of Interest

The authors declare that the research was conducted in the absence of any commercial or financial relationships that could be construed as a potential conflict of interest.
